# Impact of Donation Mode on the Proportion and Function of T Lymphocytes in the Liver

**DOI:** 10.1371/journal.pone.0139791

**Published:** 2015-10-29

**Authors:** Emmanuel Xystrakis, Muhammed Yuksel, Fang Lin, Xiaohong Huang, Oltin Tiberiu Pop, Alberto Quaglia, Nigel Heaton, Andreas Prachalias, Mohamed Rela, Susan Fuggle, Yun Ma, Wayel Jassem

**Affiliations:** 1 Institute of Liver Studies, Faculty of Life Sciences and Medicine, King’s College London at King’s College Hospital, London, United Kingdom; 2 Nuffield Department of Surgical Sciences, John Radcliffe Hospital, University of Oxford, Oxford, United Kingdom; The University of Melbourne, AUSTRALIA

## Abstract

**Background:**

Liver T-cells respond to the inflammatory insult generated during organ procurement and contribute to the injury following reperfusion. The mode of liver donation alters various metabolic and inflammatory pathways but the way it affects intrahepatic T-cells is still unclear.

**Methods:**

We investigated the modifications occurring in the proportion and function of T-cells during liver procurement for transplantation. We isolated hepatic mononuclear cells (HMC) from liver perfusate of living donors (LD) and donors after brain death (DBD) or cardiac death (DCD) and assessed the frequency of T-cell subsets, their cytokine secretion profile and CD8 T-cell cytotoxicity function, responsiveness to a danger associated molecular pattern (High Mobility Group Box1, HMGB1) and association with donor and recipient clinical parameters and immediate graft outcome.

**Results:**

We found that T-cells in healthy human livers were enriched in memory CD8 T-cells exhibiting a phenotype of non-circulating tissue-associated lymphocytes, functionally dominated by more cytotoxicity and IFN-γ-production in DBD donors, including upon activation by HMGB1 and correlating with peak of post-transplant AST. This liver-specific pattern of CD8 T-cell was prominent in DBD livers compared to DCD and LD livers suggesting that it was influenced by events surrounding brain death, prior to retrieval.

**Conclusion:**

Mode of liver donation can affect liver T-cells with increased liver damage in DBD donors. These findings may be relevant in designing therapeutic strategies aimed at organ optimization prior to transplantation.

## Introduction

Liver transplantation is the only treatment for acute and chronic liver failure. However, extending criteria to include more patients for transplant, and the persistent global organ shortage have increased the median waiting time and mortality on the waiting list [1].

The increased demand for organs has led to utilization of marginal donor, which include deceased donors after cardiac death (DCD), older donors and those with abnormal liver biochemistry. Marginal organs are vulnerable and have been seen to be associated with increased rate of graft failure following transplantation [2]. There is increasing evidence that brain death (BD) has a number of sequelae on donor organs, impacting on graft and patient outcome [3].

Animal and clinical studies have demonstrated that BD induces haemodynamic instability, hormonal deregulation, release of cytokines and chemokines, up-regulation of adhesion molecules and increase in leucocyte infiltration predisposing allografts to increased ischemia/reperfusion injury (IRI), and increasing their primary dysfunction and rejection rates [4] [5] [6] [7] [8]. Currently there is no specific therapy to treat the effects of BD and studies aiming at reducing the level of transaminase and the rate of acute rejection with steroid pre-treatment of the donor did not improve outcomes after liver transplantation [9].

Liver is considered a lymphoid organ and harbours abundant numbers of T-cells that are important for hepatic defence against antigen and gut bacteria [10]. The characterisation of intrahepatic lymphocytes has been elucidated in animal and clinical studies [11]. However, the impact of BD on intrahepatic lymphocytes is unknown.

It has been well defined that innate immunity is involved in the process of IRI as it was described by Kruger et al in the setting of human kidney transplantation that TLR4 and its ligand, HMGB1, could induce more tissue damage following I/R [12]. Interestingly, they also reported donor mode could influence the expression level of TLR4 and HMGB1, being higher in donor kidneys from deceased than from living donors. However, the link between T-cell response and intensity of innate immunity in the context of donor mode has not been defined yet.

Here, we sought to investigate a liver-specific pattern in T-cell populations using liver perfusate obtained from healthy living donors (LD), DBD and DCD livers and to examine whether pre-retrieval events could alter their proportion, function and responsiveness to HMGB1.

## Materials and Methods

### Isolation of hepatic and peripheral blood mononuclear cells

Deceased donor livers (21 DBD, 12 DCD) were *in situ* perfused with preservation solution. Following organ removal, livers were additionally flushed *ex situ* through the artery and portal vein prior to cold storage. Additionally, partial livers obtained from 22 LD, were *ex situ* perfused following removal. University of Wisconsin (UW) preservation solution was used for the flash out and cold storage. The key difference between DCD and DBD is that DCD grafts undergo a prolonged period of warm ischemic time prior to cold perfusion. Warm ischemia is the period the starts when the systolic blood pressure in the donor is below 50 mmgH to the time of cannulation and cold perfusion. Liver perfusates were collected from consecutive transplants, at the end of cold storage during pre-transplant preparation [13]. Approximately 40 to 60 x10^6^ living hepatic mononuclear cells (HMC) were isolated using density gradient centrifugation (Lymphoprep, GE, Sweden).

The same method was used to isolate PBMC from 20ml of whole blood from LD.

For collection of tissue HMC, unused segments of livers, which consisted of discarded segments 4 from a split procedure were mechanically disrupted and incubated with a solution of collagenase P (Roche, UK) and DNase (Sigma) [14]. The released cells were processed as the perfusate. This study was approved by the Ethics Committee of King’s College Hospital. Furthermore, written informed consent from all the donors or their next of kins was obtained for the use of liver perfusate and blood sample in research described in this investigation.

Graft performance was assessed by aspartate aminotransferase (AST), international normalised ratio (INR) and bilirubin levels one week after transplantation.

### Cell surface staining

Total HMC were suspended in PBS with 10% FCS (PAA, UK) prior to antibody incubation to make 0.5x10^6^ cells per combination, then the cells were stained for 20 min at 4°C. The following antibodies were used: **FITC**: CD127, CD45RA, CD103, **PE**: CD25, CD69, CD49d, **PercP Cy5.5 or Pe-Cy5**: CD8, CD45RO, CD107a **APC**: CD8, CD19, CD62L, CD56, **APC-Cy7**: CD4 and **PE-Cy7**: CD3, from Biolegend (Cambridge, UK). Cells were acquired on a FACS Canto-II (BD, USA). Different cell subsets were identified by forward and side scatter and combinations of the above mentioned surface markers. Analysis was performed using Flowjo (Treestar Inc, USA).

### Intranuclear staining for FOXP3

The frequency of cells positive for FOXP3—transcription factor for Tregs—was determined by intranuclear staining after cell fixation and permeabilization using buffers from eBioscience and counter-staining with APC-conjugated anti-FOXP3 (clone PCH101, eBioscience). Flow cytometry was then performed.

### Functional analysis

For detection of differentiated T-cell subsets a minimum of 10x10^6^ HMC or PBMCs were stimulated with a cocktail of PMA/Ionomycin/Brefeldin (eBioscience, UK) for 4h, fixed (Cytofix/CytoPerm eBiosciences) and stained for cytokine detection. The antibodies used (**FITC**: IL-6, IFN-γ, **PE**: IL-17, IL-23, IL-22, **APC**: TNF-α, IL-10, IL-2) were from Biolegend. 7AAD was used to exclude dead cells from analysis.

For assessment of cytokine production in culture, total HMC (adjusted to contain 1x10^6^ T-cells), were stimulated with anti-CD3/CD28 coated beads (Life Technologies, UK) for 24 hours or left unstimulated in RPMI with 10% FCS and antibiotics (PAA). Supernatants were analysed for cytokine concentration with a cytometric Th1/Th2/Th17 bead array (BD, UK) as per manufacturer instructions.

For the study of HMGB1, liver DC isolated by negative selection (Pan-DC enrichment, Miltenyi) and autologous liver CD8 T-cells isolated by negative selection (untouched human CD8 T-cells, Invitrogen) at the ratio of 1:20 were stimulated by human rHMGB1 at 10ng/ml and 100ng/ml for 7 days (R&D systems).

### Flow cytometry based CD8 T-cell cytotoxicity assay

Carboxyfluorescein succinimidyl ester (CFSE) 5μM (final concentration, Sigma-Aldrich, UK) loaded K562 cells (Sigma-Aldrich, UK) were co-cultured, in RPMI with 10% FCS, at 37°C, 5% CO_2_ incubator at E:T ratio = 20:1, with liver mononuclear cells retrieved from a separate cohort of liver perfusates from 6 DBD and 5 DCD liver donors. After 4 hours, the percentage of remaining (CFSE+) K562 cells and the percentage of CD107a expression on gated CD8+ T-cells was analysed by flow cytometry (FACS Canto-II).

### Histology and immunohistochemistry

Tissue samples (pre- and one hour post-reperfusion) obtained from 5 DBD and 6 DCD livers were included in the study. Four μm sections were obtained from each formalin-fixed, paraffin-embedded sample and stained with H&E. For single epitope immunohistochemistry we used an anti-HMGB1 antibody (polyclonal, rabbit anti-human; Abcam; 1:1000 dilution) and the EnVision^™^ G/2 Doublestain System, Rabbit/Mouse (DAB+/Permanent Red, Dako), with 3,3’-diaminobenzidine for signal detection. Immunostains were analysed by a liver histopathologist (AQ) who was blinded to the clinical data.

### Statistical Analysis

The normality of frequency of the means of values was tested and appropriate one way ANOVA or Kruskal Wallis two-tailed tests were used to examine statistical significance (GraphPad Prism, La Jolla, USA). For correlation analysis between clinical variables and the laboratory findings, a non-parametric two-tailed bivariate test (IBM-SPSS software) was used. When two groups were compared, Student t test or Mann-Whitney U test was performed for statistical analysis (GraphPad Prism).

## Results

### Donors and recipients

There were no statistically significant differences between DBD, DCD and LD donors regarding age, gender and body weight (Table 1). Cold ischemia time was similar between DBD and DCD but significantly shorter for LD donors (Table 1). All recipients were patients with stable chronic liver disease (Table 2) and received immunosuppressive therapy (tacrolimus, prednisolone).

**Table 1:** Clinical parameters of liver donors.

Variables	DBD	DCD	LD
N	21	12	22
Donor Gender (M/F)	10/11	5/7	12/10
Donor Age (years ± SD)	50 ±16	48 ± 11	40 ± 9
Donor Weight (Kg ± SD)	78 ± 17	72 ± 13	79 ± 13
Inotropic support (Yes/No)	18/21	6/12	0/22
Infection (y/n)	1/21	1/12	0/22
Cold ischemia time (minute)	520 ±156	426 ± 129	190 ± 127

**Table 2:** Clinical parameters of recipients.

Variables	DBD	DCD	LD
n	21	12	22
Recipient Age (years ± SD)	47 ± 16	53 ± 11	22 ± 26
Rejection (biopsy proven) (two-week post-transplant)	1/21	2/12	2/22
Recipient AST peak 1^st^ week (IU ± SD)	1279 ± 375	3920 ± 907	448 ± 340
Recipient total bilirubin peak 1^st^ week (mg/dl ± SD	48 ± 39	79 ± 54	55 ± 33

### Hepatic CD8 T-cells exhibit memory phenotype and are mostly abundant in DBD donors

**Fig 1:**
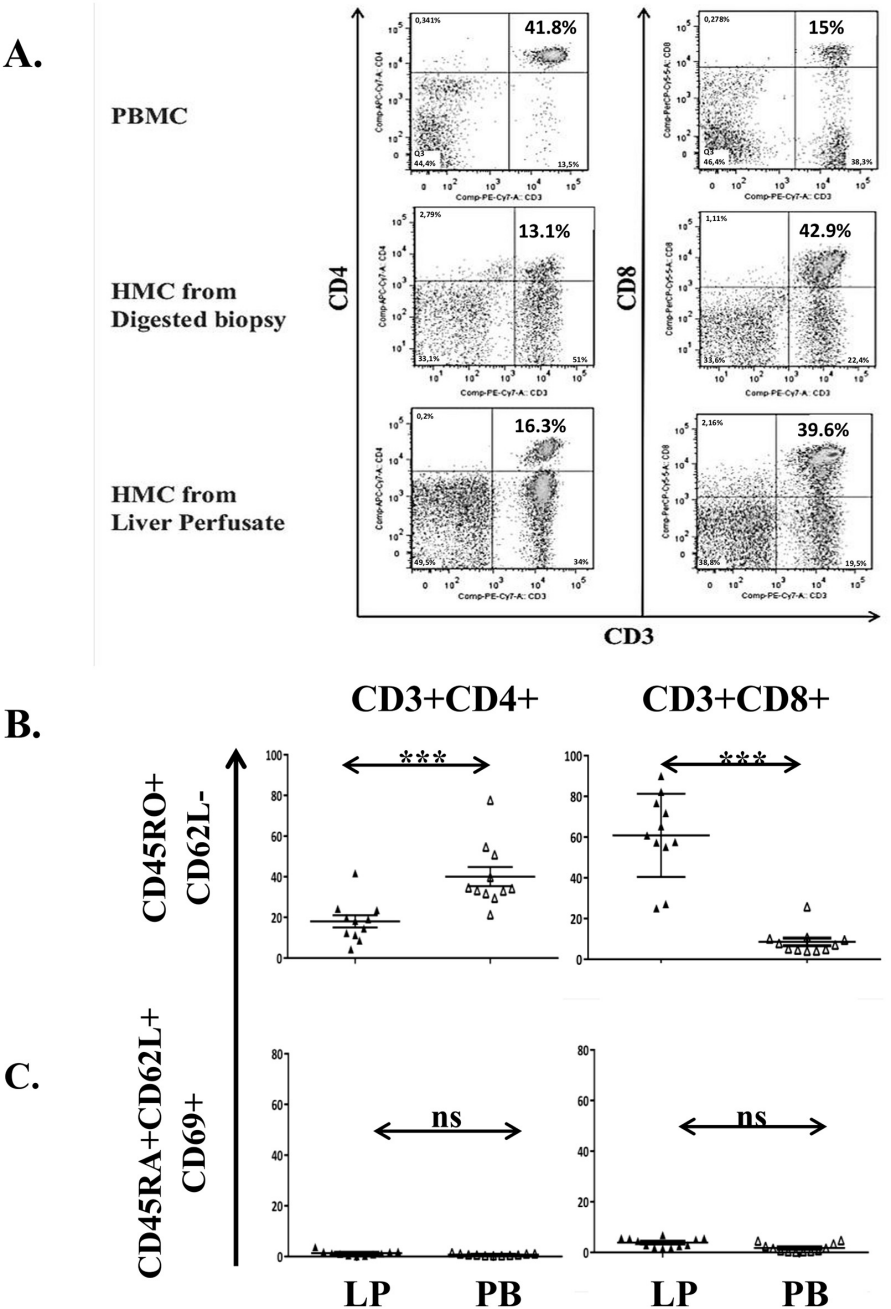
The dominant T-cell subset of the liver is memory CD8 T-cells. Hepatic mononuclear cells (HMC) isolated from liver perfusate of living donors (LD, n = 11) were compared to matched PBMC. (A) Dot plots of CD4 and CD8 T-cells isolated from blood, through collagenase digestion and perfusion of the same liver. (B) Analysis of the combined expression of CD3, CD4, CD8, CD45RO and CD62L; on CD4+ (left) and CD8+ (right) T-cells (Mann-Whitney U test); on (C, D) CD4 (left) and CD8 (right) CD45RA+CD62L+ naïve (C) and CD45RO+CD62L- memory (D) CD69+ T-cells (Mann-Whitney U test); (E) CD127 expression on memory CD45RO+CD62L- CD4 (left) and CD8 (right) T-cells (Student t-test). ns = non-significant, *** p<0.001, ** p<0.01.

First we confirmed that liver perfusate (LP) is a representative source for liver-associated T-cells by comparing the phenotype of cells isolated from LP, liver biopsy (Fig 1A) and peripheral blood (PB) from the same donor. Viability of the cells isolated from the LP was constantly higher than 95% and the analysis showed that LP derived from LD contained a lower frequency of CD4 (19.7±1.5% vs 38.8±2.6%) but higher CD8 (41.8±1.6% vs 19.3±1.7%) than that from PB.

Further analysis comparing T-cells from LP of LD and PB showed that T-cell subsets in LP contained a significantly higher proportion of memory CD45RO+CD62L-CD8+ T-cells compared to PB (60.8±6.0% vs 10.4±3.2%) but the frequency of memory CD45RO+CD62L-CD4+ T-cells was lower (16.5±3.1% vs 41.0±5.7%, Fig 1B). Liver but not PB T-cells were found expressing the early activation marker CD69 [15] and its expression was within the CD45RO+ memory compartment (Fig 1C and 1D). Additional phenotypic analysis of these CD45RO+CD62L-CD8+ and CD45RO+CD62L-CD4+ liver T-cells using CD127, CD25, CD103 and CD49d markers [16] [17] [18] [19] [20], showed that the proportion of memory CD127 positive CD8 T-cells was higher than that in PB (42.7±4.0% versus 16.7±2.5%), however, less CD4 T-cells expressed CD127 compared to PB (23.4±3.3% vs 57.8±5.7%, Fig 1E). Memory CD8 but not CD4 liver T-cells also co-expressed CD103 with CD69 albeit only on 20% of memory CD69+CD8+ T-cells (not shown). We did not detect significant expression of CD49d on any subset of liver T-cells.

**Fig 2:**
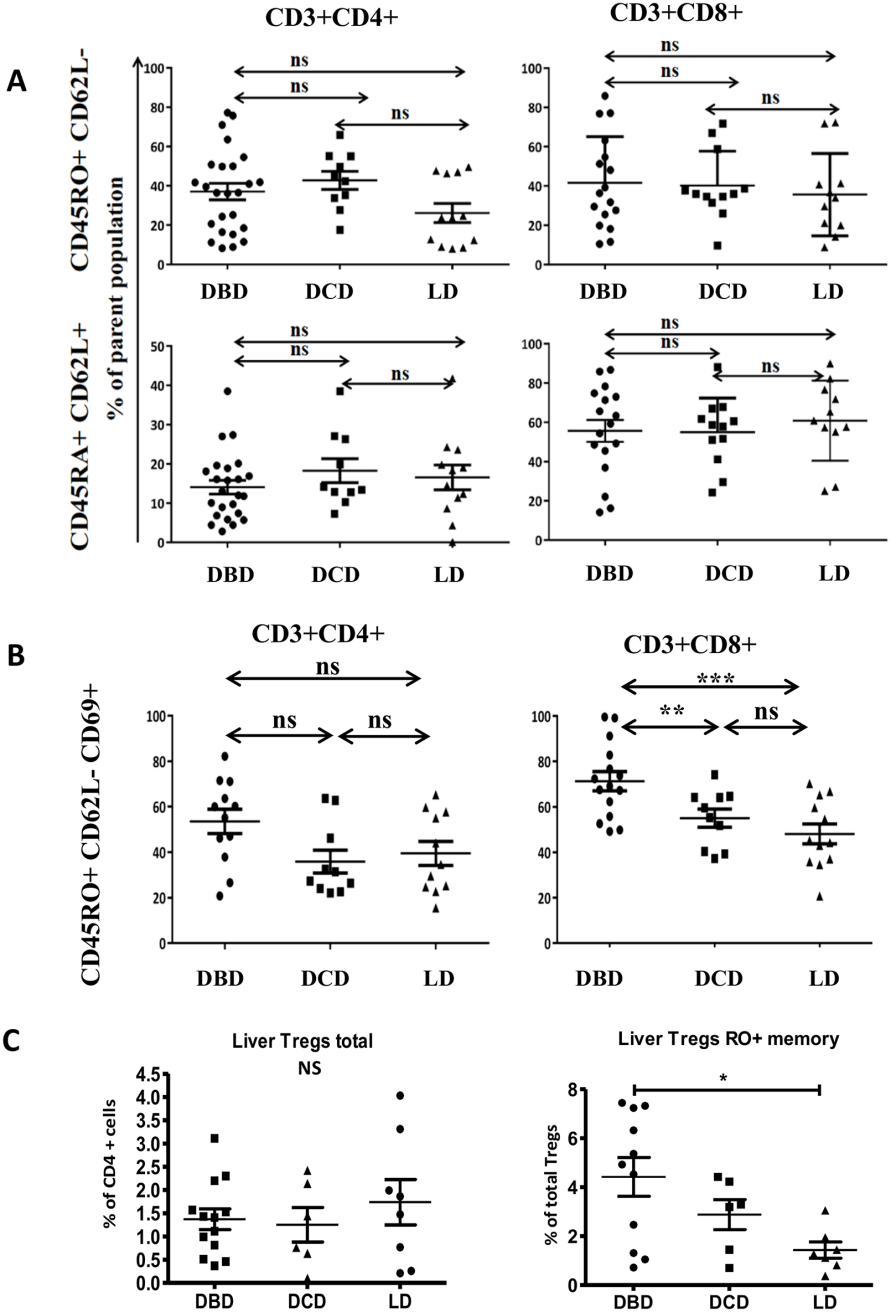
CD8 memory T-cells expressing CD69 are increased in DBD livers. (A,B,C) analysis of HMC isolated from DBD (n = 12 to 26), DCD (n = 5 to 10) and LD (n = 8 to 11) livers were assessed for combined expression of CD3, CD4, CD8, CD45RO, CD45RA, CD62L, CD69 and FOXP3. Kruskal Wallis test was used for (A and B), one-way ANOVA was used for (C). Frequency of CD4+ (left) and CD8+ (right) T-cells in the perfusate of indicated donors. ns = non-significant, *** p<0.001, ** p<0.01, * p<0.05.

We then investigated whether the mode of donor death altered the proportion of liver T-cells by comparing the proportion of CD4 and CD8 T-cells obtained from DBD, DCD and LD livers. The frequency of memory and naïve T-cell subsets was similar among different donor groups (Fig 2A). CD69 expression on memory CD4 T-cells was similar on all donors (LD = 39.5±5.1%, DBD = 53.5±5.3%, DCD = 35.8±5.0%), but the frequency of CD69+CD8 memory T-cells was significantly higher in DBD than in DCD and LD (71.2±4.2%, 55.3±3.9% and 48.1±4.4% respectively, Fig 2B). Furthermore, Memory RO+ Tregs, but not total Tregs were more frequent in DBD compared to DCD and/or LD (Fig 2C).

### The function of liver-associated T-cells are dependent on the donation status of the allograft

**Fig 3:**
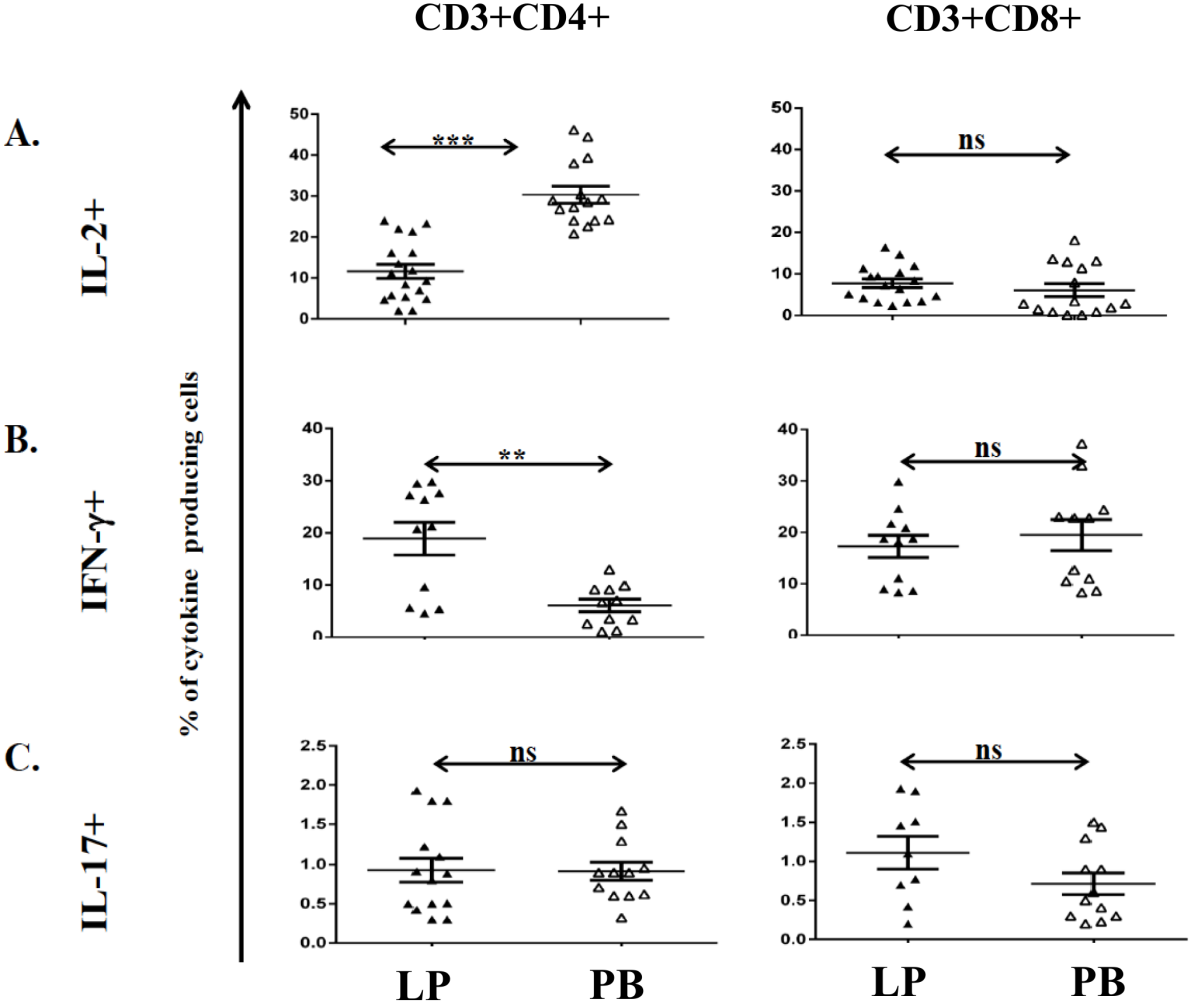
Cytokine production of T-cells in peripheral blood, LD, DBD and DCD livers. Hepatic mononuclear cells isolated from liver perfusate of living donors (LD) and matched PBMC were stimulated *ex vivo* and their cytokine production (n = 9 to 17) was assessed by intracellular staining. Frequency of IL-2 (A), IFN-γ (B) and IL-17 producing CD4 (left) and CD8 (right) T-cells (C). ** p<0.01, *** p<0.001. Furthermore, HMC isolated from DBD, DCD or LD livers have been stimulated *ex vivo* and cytokine production assessed by intracellular staining. Gating strategy determined using unstimulated cells. Numbers indicate percentage of cells detected; (D-F) Cytokine production of HMC isolated from indicated liver donors. Frequency of CD4 or CD8 T-cells expressing IL-2 (D), IFN-γ, E) and IL-17 (F). Statistical analysis: Mann Whitney U or Kruskal Wallis test was applied, ns = non-significant, **p<0.01, ***p<0.001.

We then compared the ability of liver and circulating T-cells to produce cytokines after PMA/ionomycin stimulation. Hepatic CD4 T-cells produced less IL-2 (11.6±1.7% vs 30.3±2.0%, Fig 3A) but significantly more IFN-γ (18.9±3.1% vs 6.1±1.2%, Fig 3B) than that by PB. There was no difference in the frequency of IL-17 producing T-cells between liver and blood (Fig 3C). There was also no significant difference between liver and circulating CD8 T-cells in terms of IL-2 and IFN-γ production (7.7±1.1% vs 6.1±1.5% and 17.2±2.1% vs 19.5±3.0% respectively, Fig 3B). Only in a small number of donors (n = 6, not shown) liver T-cells produced TNF-α and IL-10 in a significantly lower frequency than blood. IL-4, IL-6, TGF-β and IL-22-producing T-cells were undetectable.

Further analysis showed that the mode of donation affected the function of liver T-cells: CD4 but not CD8 T-cells were the major IL-2 producers (DBD = 14±1.8%, DCD = 10.7±1.6% and LD = 11.6±1.7%, Fig 3D). IFN-γ-production was mostly seen by DBD CD8 T-cells (DBD = 31.3±1.8%, DCD = 8.6±2.7% and LD = 17.2±2.1% Fig 3E) and IL-17-production by DCD CD4 T-cells (DBD = 1.4±0.2%, DCD = 2.7±0.3% and LD = 0.9±0.1%, Fig 3F). The frequency of IFN-γ (Fig 3B) or TNF-α-producing (not shown) CD4 T-cells was not statistically significant different among donor groups.

**Fig 4:**
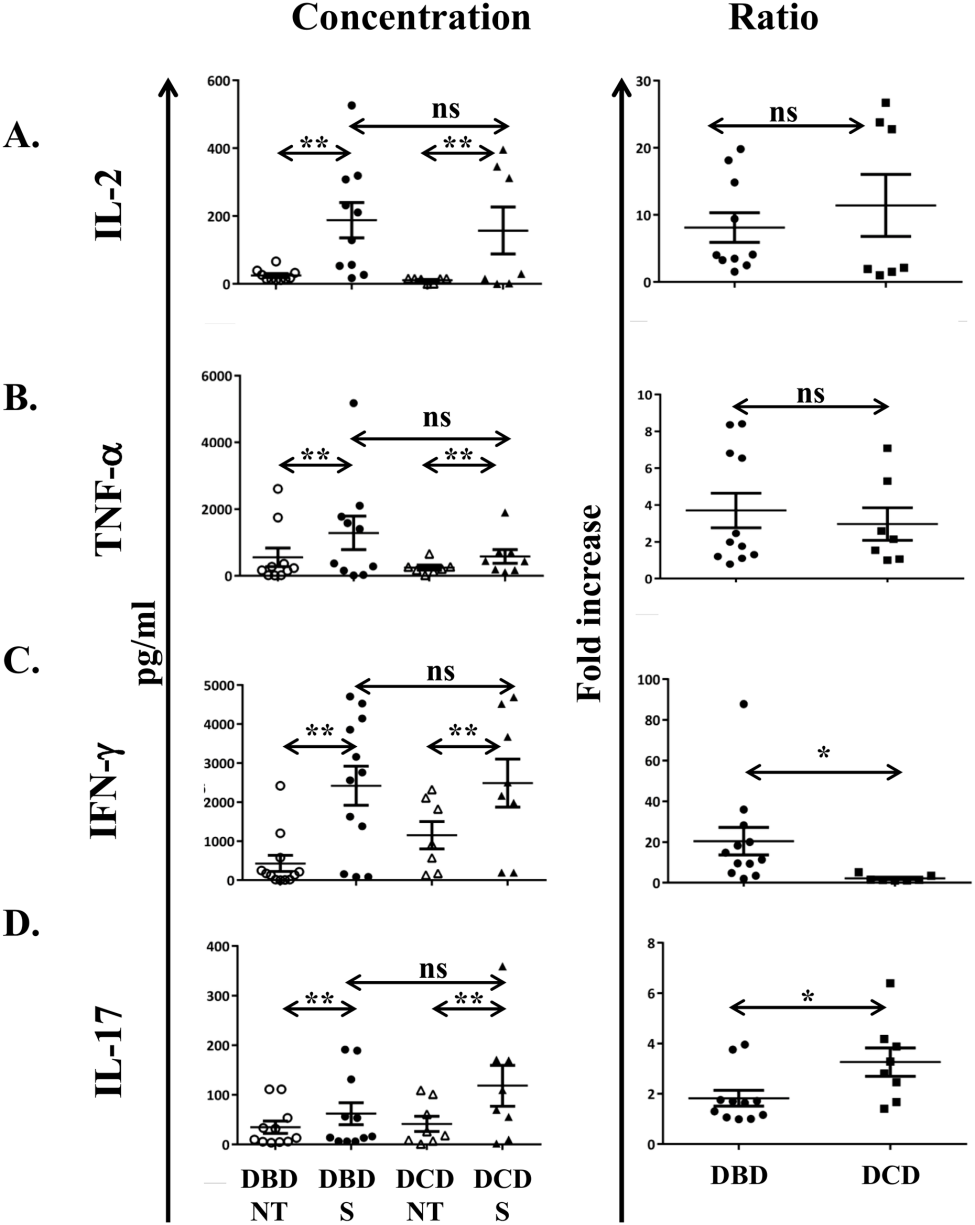
The donation status of the liver allograft influences the cytokine production in culture. Cytokines in the supernatants of day-seven HMC culture were measured. Left column: levels of IL-2 (A), TNF-α (B), IFN-γ(B), IFN-in the supernatants of day-seven HMC culture were measured. Left column: levels of IL-2 (A), TNF-ells. Numbers indicate epatocellular injury iα (B), IFN-γ(B), IFN-in the supernatants of day-seven HMC un-stimulated and stimulated HMC. Mann Whitney U or Kruskal Wallis test was performed. ns = non-significant, * p<0.05, **p<0.01, ***p<0.001.

It is arguable that cytokine production after PMA/Ionomycin stimulation is not physiological. To confirm our observations, we cultured total HMC isolated from DBD and DCD livers and assessed the cytokines in the culture supernatant. We detected high levels of IL-2, IFN-γ and TNF-α in both groups of donors (Fig 4A and 4B) and showed that the fold increase in IFN-γ production was significantly higher in DBD donors (Fig 4C), while IL-17 was increased significantly in DCD donors (Fig 4D), similarly to PMA/Ionomycin stimulation.

### Liver CD8 T-cells respond indirectly to HMGB1 *in vitro*

**Fig 5:**
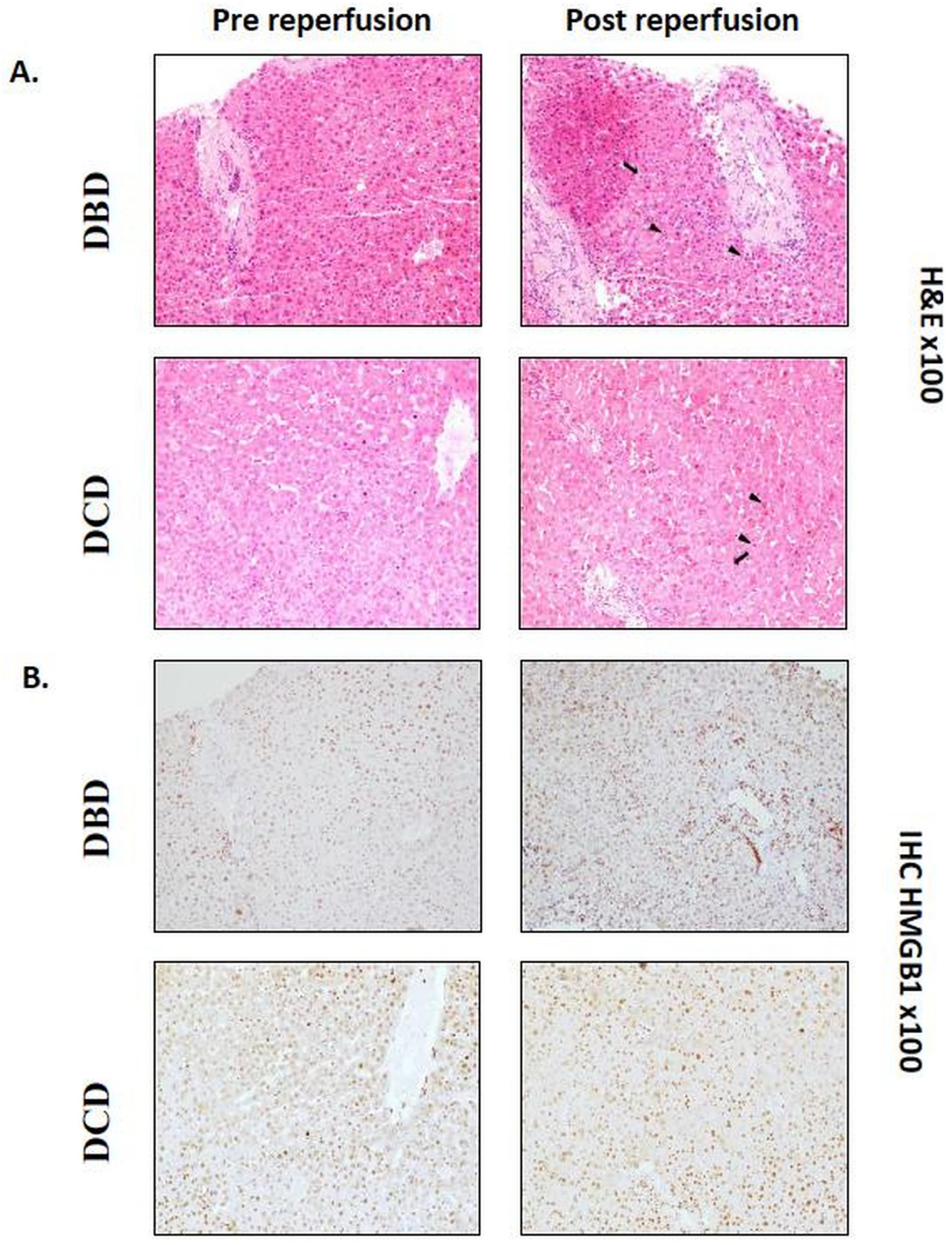
Intrahepatic T-cells respond to HMGB1 *in vitro*. (A, B) Biopsies obtained from liver allografts before (left) and one-hour after (right) reperfusion. (A) Haematoxylin and eosin staining of a representative biopsy from DBD or DCD livers showing hepatocellular necrosis (plain arrow) and neutrophil aggregation (arrowhead) in post-reperfusion samples; (B) Immuno-histochemical staining for HMGB1 of the biopsies shown in (A) reveals an increased proportion of hepatocytes showing nuclear expression of HMGB1 in post-reperfusion samples compared to the pre-reperfusion counterparts but no cytoplasmic expression; (C) Production of IFN-γ from T-cells of a representative donor co-cultured with syngeneic DC at the ratio of 20:1 for 7 days. Percentage of IFN-γ-producing cells gated on CD8+CD3+ T-cells in unstimulated cultures (negative control), in presence of anti-CD3/CD28 (positive control) or in presence of HMGB1 at 100ng/ml; (D) Proliferative response of T-cells in unstimulated cultures (negative control), in presence of anti-CD3/CD28 (positive control) or in presence of HMGB1 at 100ng/ml. 1 = negative, 3 = positive control and 2 = HMGB1; (E) Quantitative analysis of IFN-γ concentration in supernatants of cultures. NT = negative control, S = aCD3/CD28 stimulation, HMGB1 = cultures stimulated in presence of 100 or 10ng/ml of HMGB1. Depiction of IFN-γ concentration in pg/ml (left) and ratio of increase reported to the negative control (right). Kruskal Wallis test was used.

In order to assess whether HMGB1 is differentially expressed in biopsies of DBD and DCD livers, immunohistochemistry was performed on pre- and post-reperfusion biopsies where histological signs of IRI changes such as foci of hepatocyte necrosis and neutrophil aggregates were detected (Fig 5A). Immuno-histochemical staining for HMGB1 showed intranuclear expression in hepatocytes, biliary epithelium and inflammatory cells (Fig 5B). Although the frequency of HMGB1-expressing hepatocytes was increased in some [2/5 (40%) DBD and 3/6 (50%) DCD] of the post-reperfusion samples compared to their pre-reperfusion counterpart, there was no significant correlation with the magnitude of IRI lesions or the donation status of the allograft. In addition, no hepatocyte cytoplasmic staining was identified in the post-reperfusion samples.

Functional analysis of the effect of rHMGB1 on hepatic T-cells in co-cultures with autologous DC showed that addition of rHMGB1 induced increase in IFN-γ-producing CD8 T-cells (Fig 5C), a proliferative response (Fig 5D) and increased secretion of IFN-γ in the culture supernatants of DBD and DCD liver CD8 T-cells (Fig 5E) compared to the unstimulated control, suggesting that hepatic T-cells are responsive to HMGB1 through the indirect effect of DC. However, the responses to rHMGB1 stimulation did not differ between DBD and DCD conditions.

### Hepatic CD8 T-cell CD107a expression

**Fig 6:**
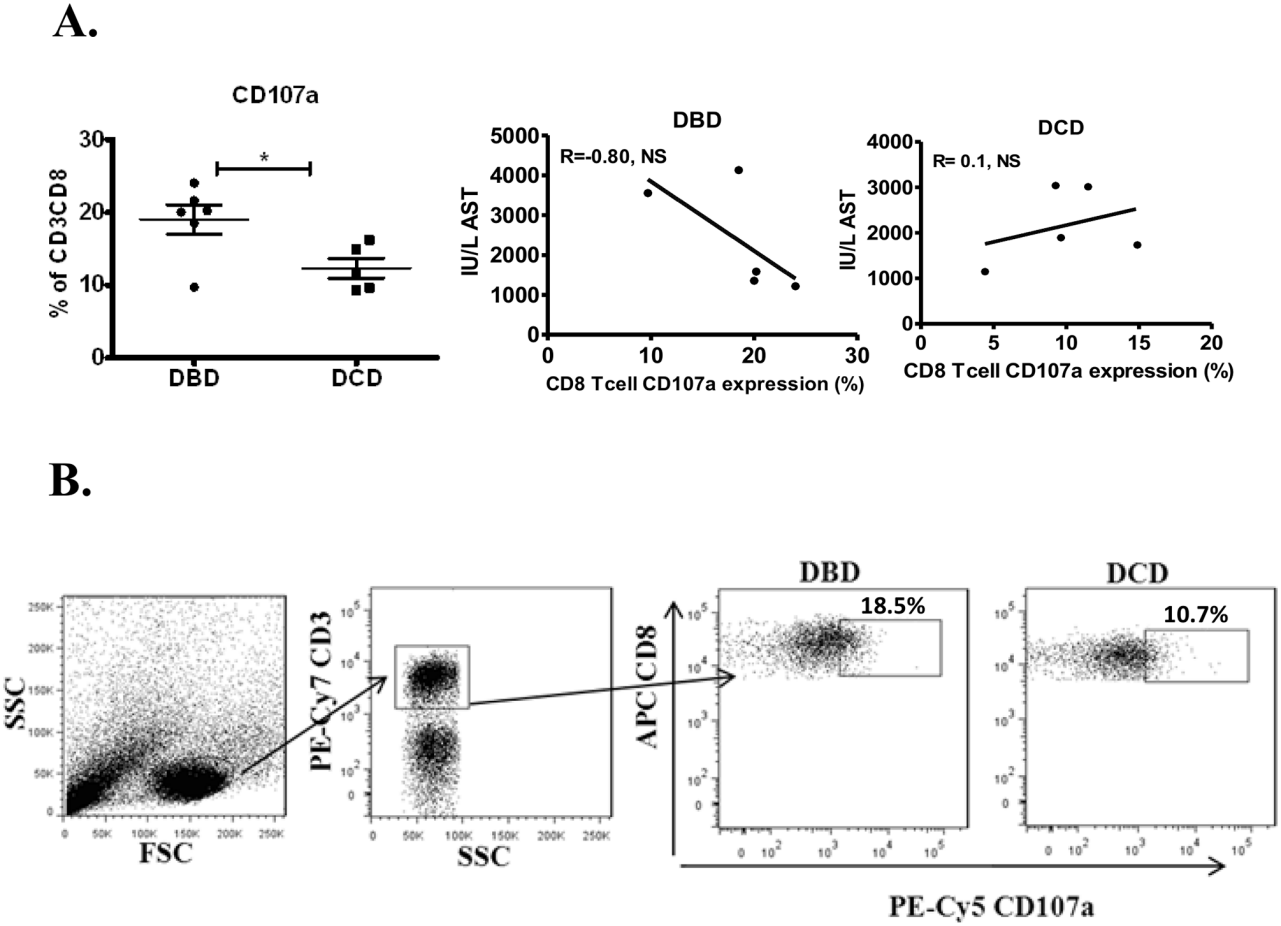
CD107a expression in liver isolated CD8+ T-cells. (A) In vitro CD107a expression on liver (DBD and DCD) CD8+ T-cells was analysed after a co-culture with K562 cell line. Mann-Whitney U or Spearman correlation test was used (B) Gating of liver mononuclear cells to analyse CD107a expression on CD8+ T-cells. ns = not significant, * p<0.05.

The percentage of K562 killing was similar for both donation modes exceeding >90% killing, likely a consequence of NK cell activity to which K562 cell line is vulnerable to. Furthermore, cytotoxic degranulating CD8 T-cells upregulate their cell surface CD107a expression and as such is a marker for their cytotoxicity function [21]. Since CD8+ T-cells produced significantly more IFN-γ after PMA/Ionomycin stimulation, this prompted us to investigate the expression of CD107a on CD8+ T-cells, gated on CD3+ T-cells (Fig 6A), isolated from liver perfusates of DBD and DCD donors after co-culture with K562 cell line. We found that CD8+ T-cells from DBD liver donors expressed significantly more CD107a compared to DCD liver donor CD8+ T-cells (Fig 6B). Next, we questioned whether this upregulation was associated with episodes of acute rejection or one-week post-transplant graft performance. No significant changes were detected, however, a trend of negative correlation (r = -0.8, p = 0.2) between CD107a expression and peak of AST levels in DBD donors was observed, whereas in DCDs no association or trends were detected (Fig 6A).

### Correlation with clinical variables

We examined the association between laboratory findings with donation mode and recipient’s clinical data. Among the recipients of DBD livers, there were positive correlations between the frequency of memory CD8 T-cells (r = 0.55 p<0.05), CD69+CD8 T-cells (r = 0.63 p<0.01) and IFN-γ-producing CD8 T-cells (r = 0.55, p<0.05) and AST peak levels. There was a negative correlation between IL-10 producing CD4 T-cells and the duration of cold ischemia (r = -0.63, p<0.01). There was no correlation with the frequency of IL-17-producing CD4 T-cells. Among the recipients of DCD livers, there were positive correlations between memory CD8 T-cells and the peak of bilirubin level (r = 0.87, p<0.01) and also the warm ischemia time (r = 0.78, p<0.01).

## Discussion

The main findings of this study are, first, intrahepatic lymphocytes are mainly represented by IFN-γ-producing memory liver-associated CD8+ T-cells; second, the proportion and function of CD8+ T-cells were affected by the mode of death in the donor and correlated with immediate graft outcome; third, CD8+ T-cells obtained from DCD and LD livers are less activated when compared to DBD.

Our data are in agreement with the previous report, where a comprehensive description of the intrahepatic proportion of T-cells in humans has been achieved by isolation of hepatic mononuclear cells by sequential perfusions of LD livers [15]. This study showed a widespread expression of various activation markers, including CD69, on all T-cells suggesting a continuous state of activation, perhaps through the permanent flux of exogenous antigens from the portal circulation. Similar studies on animals have shown that tissue-derived inflammatory signals promoted the survival of memory CD8+ T-cells [22], thus explaining the presence of functionally-active CD8+ T-cells in the liver following vaccination [23] or infection [24], and in the current study following the process of donation.

In the liver, it has been suggested that T-cells express CD69 in response to activation by gut-derived bacterial antigens to provide a state of tonic stimulation and increase their protective role [25]. Our findings support this suggestion, since CD69 expression is mainly absent on naïve hepatic T-cells but present on a proportion of memory CD8 T-cells. This conclusion is further supported by analysis of the activation state of liver-associated CD8+ T-cells. The combination of high CD127 and low CD25 expression together with their cytokine profile, a robust IL-2 and IFN-γ production, correlates with their surface phenotype.

The correlation between levels of CD69-expressing CD8+ T-cells and IFN-γ production with the early peak of AST in recipients of DBD grafts, the warm time and bilirubin levels following in DCD organs indicates the implications of these cells in liver injury. These findings are in agreement with previous animal data demonstrating that the induction of brain death induces an inflammatory process, enhancing IRI [26].

In contrast, liver-associated CD4+ T-cells preferentially express the naïve cell marker CD45RA, a feature that was not predicted by other studies where a majority of memory CD4+ T-cells was found [27]. Their high CD69 expression and preferential production of IFN-γ indicate an activated terminal-effector phenotype, which is in contrast to their preference to express CD45RA. However, it has been proposed that terminally differentiated effector-memory T-cells may up-regulate the expression of CD45RA [28]. Interestingly, liver-associated CD4+ T-cells contain a higher number of IL-17 producing T-cells in DCD allografts than in DBD and LD. This may be related to the extended period of warm ischemia in DCD. This finding is similar to that seen in a mouse DCD model, in which hepatic CD4+ T-cells produced a high level of IL-17 [29].

Although, CD69 is well accepted as an activation marker, the high expression pattern of CD69 by LP, but not by PB, memory CD4+ and CD8+ T-cells suggest that CD69 is a marker for tissue associated T-cells. Indeed, in a recent publication, Mackay et al showed that CD69 is involved in the prolonged retention of tissue T-cells by down regulating the sphingosine-1 phosphate receptor [30].

By studying three types of liver donors, including DCD, we showed that patterns of T-cell proportion may vary according to the donation status, suggesting that the mode of organ donation may influence the homing potential of T-cells. Although the reasons for the retention of these cells in the healthy hepatic tissue are not clear, the increased proportion of memory CD8+IFN-γ producing T-cells in the DBD livers suggests that non-specific inflammatory signals related to brain death could be involved as it has been demonstrated in a mouse model exploring the effects of brain death in the adaptive immunity [31]. These findings are supported by our previous studies where liver biopsies taken before transplantation showed increased inflammatory changes, such as up-regulation of ICAM-1 and neutrophil infiltration, in DBD donors compared to LD which were associated with infections and use of inotropes in the donor [32]. Furthermore, the upregulation of CD107a expression on CD8 T-cells in DBD livers displayed a trend of negative correlation with the post-transplant AST. This finding was unexpected. There might be two explanations, first, these CD107a expressing CD8+ T-cells may be exhausted T effectors by co-expressing the programmed cell death-1 (PD-1), the exhaustion marker. One might assume that exhausted T-cells will induce less damage to the allograft; second, as described by Steinert E et al in a recent mouse study [33], the tissue resident memory T cells (T_RM_) in non-lymph tissue appear to have the strongest immune function to eliminate pathogens. CD107a+ CD8+ T-cells could form one subset of T memory cells in the liver and those secreting IFN-γ might represent the cell subset causing severe allograft injury. Nonetheless, more investigation is necessary to characterise further these CD107a+ CD8+ T-cells.

In additional to cytotoxicity executed by CD8+ T-cells, hepatic NK cells are highly possible to be involved in the killing of K562 cells, being >90% (data not shown). Hence, the impact of donor mode on specific subsets of NK cell, such as CD161+ IL17 producing NK cells, and other cells of innate immune system warrants further investigation.

The finding that CD8+ T-cells appear less activated in DCD is of interest. This finding may be explained by the fact that organs from DCD donors are less exposed to inflammatory changes associated with brain death. These findings are supported by previous reports in liver and kidney transplantation [34]. Liver biopsies taken from DCD allografts showed reduced involvement with inflammation as demonstrated by less up-regulation of ICAM-1 and neutrophil infiltration compared to DBD allografts [32]. Additionally, biopsies obtained from kidneys before transplantation showed high levels of lymphocyte infiltration in DBD compare to DCD and LD kidney [35].

Various molecules, including HMGB1, passively released by dying cells or actively by effectors of innate immunity are potential candidates to provide the link between innate and adaptive immunity [36] [37]. We showed that HMGB1 does not translocate to the cytoplasm or the intercellular space, contrary to data obtained in animal models of liver IRI, despite studying biopsies exhibiting evident IRI-related lesions in post-reperfusion samples. This discrepancy could be attributed to the timing of post-reperfusion biopsies occurring significantly earlier than in controlled animal models or the shorter (or absent) warm ischemia time, a major cause inducing secretion of HMGB1, compared to the experimental IRI. Nevertheless, we demonstrated that HMGB1 used in cultures in concentrations equivalent to those measured in the supra-hepatic vein after reperfusion [38], is a potential activator of hepatic T-cells poised to secrete IFN-γ. We have demonstrated a close link between liver-associated T cells and innate immunity represented by HMGB1, though we could not find difference in impact of donor mode on the responsiveness.

Overall, this study shows that livers are enriched with IFN-γ-producing memory CD8+ T-cells. These cells are activated in DBD allografts and might influence immediate post-transplant graft function after transplantation. Their influence in long term and interaction with innate immunity warrant further investigation.

## Abbreviations

DBD/DCD: donation after brain/cardiac death
LD: living donors
LP: liver perfusate
HMC: hepatic mononuclear cells
HMGB1: High Mobility Group Box 1
